# Calcium Channels in the Heart: Disease States and Drugs

**DOI:** 10.3390/cells11060943

**Published:** 2022-03-10

**Authors:** Kajol Shah, Sarah Seeley, Castin Schulz, Jacqueline Fisher, Shubha Gururaja Rao

**Affiliations:** 1 Internal Medicine at Rutgers, New Jersey Medical School, Newark, NJ 07103, USA; shahkajol94@gmail.com; 2 Department of Pharmaceutical and Biomedical Sciences, Raabe College of Pharmacy, Ohio Northern University, Ada, OH 45810, USA; s-seeley.1@onu.edu (S.S.); c-schulz@onu.edu (C.S.); j-fisher.14@onu.edu (J.F.)

**Keywords:** calcium channels, calcium ions, cardiac function, arrhythmia, calcium channel blockers

## Abstract

Calcium ions are the major signaling ions in the cells. They regulate muscle contraction, neurotransmitter secretion, cell growth and migration, and the activity of several proteins including enzymes and ion channels and transporters. They participate in various signal transduction pathways, thereby regulating major physiological functions. Calcium ion entry into the cells is regulated by specific calcium channels and transporters. There are mainly six types of calcium channels, of which only two are prominent in the heart. In cardiac tissues, the two types of calcium channels are the L type and the T type. L-type channels are found in all cardiac cells and T-type are expressed in Purkinje cells, pacemaker and atrial cells. Both these types of channels contribute to atrioventricular conduction as well as pacemaker activity. Given the crucial role of calcium channels in the cardiac conduction system, mutations and dysfunctions of these channels are known to cause several diseases and disorders. Drugs targeting calcium channels hence are used in a wide variety of cardiac disorders including but not limited to hypertension, angina, and arrhythmias. This review summarizes the type of cardiac calcium channels, their function, and disorders caused by their mutations and dysfunctions. Finally, this review also focuses on the types of calcium channel blockers and their use in a variety of cardiac disorders.

## 1. Introduction to Cardiac Calcium Channels

Mammalian hearts are four-chambered with two atria and ventricles each. Blood is pumped through in accordance with action potential generated at the sino-atrial node in the right atrium [1]. The summation of currents through the various channels generates the cardiac electrical activity [2,3]. Several types of ion channels function in cardiac cells as complexes, partnering with other proteins, and generate cardiac action potential [4,5,6]. The rate and force of contraction of cardiac myocytes are controlled by an intricate network of these channels. These channels function in multiple signaling pathways of the heart [3]. Specifically, calcium channels have an important function in the physiology and regulation of heart function [7,8,9,10]. Calcium is a central ion in this electrical activity of the heart where it connects electrical signals and contraction, while also carrying out other signaling events such as gene transcription, cellular growth processes, bioenergetics, triggering immune response, and tissue remodeling [11,12,13]. The calcium current impacts both the pacemaker activity and myocardial contraction of the heart [14]. Subtle changes in calcium channel function disrupt the plateau phase of the cardiac action potential and give rise to an irregular conduction cycle [15], leading to pathophysiological conditions involving cardiac dysfunction. Hence, cardiac arrhythmia disorders have led to the identification and characterization of several of these calcium channels in the heart [8,9,10].

The primary function of the calcium channels in the sarcolemma of cardiac myocytes is to bring calcium into the cell [16]. More specifically, the movement of calcium ions from the extracellular space into the cell is primarily through voltage-gated calcium channels [17]. The influx of calcium contributes to keeping the membrane potential more positive and the released calcium from the sarcoplasmic reticulum signals the activation of contraction [16]. This is a well-known phenomenon, called “calcium-induced calcium release” [18]. As a result, the excitation–contraction coupling process in cardiac myocytes can proceed without interruption [17]. Therefore, modulation of these channels serves as an important pharmacological target, given multiple pathologies are implicated when they present an abnormal function. Here, it is important to understand the structural and functional components of calcium channels in the heart. This review explores the types of calcium channels in the heart (L-type and T-type), their structure and function, associated disorders, and the pharmacological advances targeting cardiac calcium channels.

## 2. Types of Calcium Channels in the Cardiac Tissues

There have been six classes of calcium channels identified so far, based on the rate of their activation, pharmacologic sensitivity, and also the level of voltage activation (low or high voltage). These classes are named T, L, N, P, Q and R types [19]. N, P, Q and R types of calcium channels are predominant in the nervous system. Given their expression in the cardiomyocytes, this review will focus mainly on the L- and T-type calcium channels.

In the heart, L- and T-type are the two distinct groups of calcium channels, both essential in regulating cardiac function [3]. The nomenclature of these two types as L- and T- is based on their response to voltage. Both are voltage-gated calcium channels, but L-type channels are “long-lasting” and require a strong depolarization for their activation, whereas T-type channels are not long lasting but are “transient” [19]. A thorough understanding of the structural and functional differences, along with time and spatial expression variations between these two classes, has led to the uncovering of their contribution to cardiovascular pathologies.

**Figure 1:**
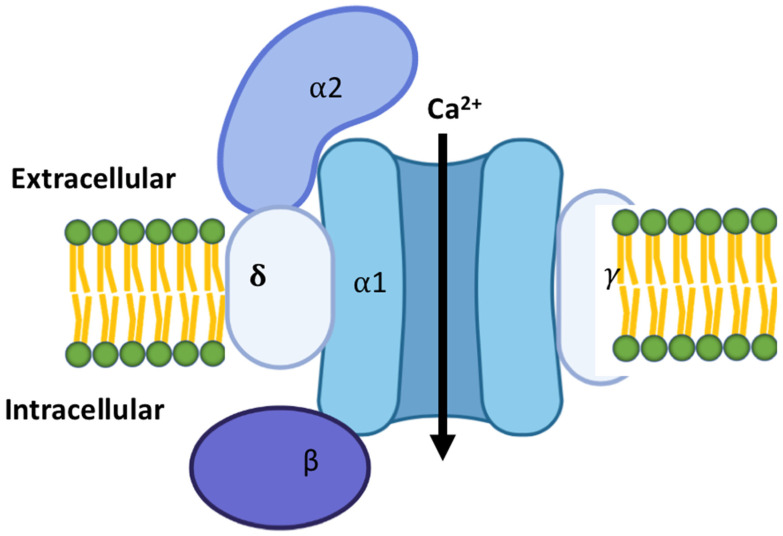
Schematic representation of the calcium channels: Calcium channels are a complex of 4–5 distinct subunits. The α1 subunit is the largest subunit and it incorporates the pore region, the voltage sensor and gating apparatus, several channel regulation sites by second messengers, drugs, and toxins. There is an intracellular β subunit, a transmembrane, α2δ subunit complex, and a transmembrane γ subunit.

L-type calcium channels are long opening and high voltage-gated channels. They are expressed in developmental as well as adult stages in the heart [19]. They are essential and are the main source of excitation–contraction coupling as an influx of calcium occurs through them during membrane depolarization [17]. They are found not only in cardiac tissue but also in skeletal muscle and all excitable cells [20]. The L-type channels are composed of four subunits [21]. These include a pore-forming subunit, alpha (α), and three accessory subunits, beta (β), delta (δ), and gamma (γ). The α1 subunit forms the pore and central transmembrane machinery, while the β subunit is located on the cytoplasmic side of the complex and interacts with intracellular domains of the α1 subunit. The γ subunit is a transmembrane protein and also interacts with the α1 subunit, in an arrangement depicted in Figure 1 [22]. These subunits function to regulate membrane trafficking, current kinetics, and gating properties [22]. The channel is structured in four repeated domains. Each of these domains consists of six transmembrane segments, S1–S6 [6]. The S5 and S6 segments from the repeats form the pore domain. The S1 to S4 segments on each of the domains form the voltage sensor for activation or inactivation states. These channels open in response to membrane depolarization [17]. The pore-forming domain may be interrupted by extracellular loops, designated as L5 or L6 [22]. This combined with different conformations of the S5 and S6 segments creates structural variability. A selectivity filter is generated by four glutamate residues on side chains and the carbonyl oxygen atoms of the two preceding residues in each repeat. A transient depolarization in cardiac cells triggers rapid contraction, directly correlating with L-type calcium channel activity [17]. This leads to a cascade of intracellular events, one being the activation of the type 2 ryanodine receptor (RyR2) [23], resulting in a slowly developing and sustained contraction [22]. The calcium current in these channels inactivates rapidly in cardiac cells compared to aortic cells [17].

L-type calcium channels have multiple functions in the heart, including regulation of early cardiac organogenesis, structural development and cardiac physiology [24]. They are highly expressed in both neonatal and adult atrial and ventricular myocytes, where they are directly responsible for the initiation of contraction with the calcium-induced calcium release [9]. They are also involved in the embryonic development of the heart as their inactivation has been shown to cause lethality [24]. Blockade of these channels early in development results in a dilated left ventricle with a thin outflow tract. These channels also contribute to cardiac contractility as activation of the channels increases chronotropic response [16]. A variety of arrhythmias including atrial fibrillation have been associated with alterations in L-type channels [16].

T-type channels open to low-voltage depolarizations and are transient in nature compared to their L-type counterparts. They are functionally expressed in embryonic hearts but their expression reduces along developmental stages [25]. In the adult heart, T-type channels are not expressed in ventricular myocytes but are mainly found in atrial or sinusoidal cells of the heart [26]. The channels activate at a more negative potential and have rapid inactivation kinetics [26]. The structure of T-type calcium channels also consists of four homologous domains along with accessory subunits [21]. The N- and C-termini are present in the intracellular region with three linkers that connect the domains [27]. The four homologous domains are composed of six transmembrane helices (S1 to S6) [21]. These domains are linked together with intracellular loops, specifically between the S6 portion of the preceding domain and the S1 portion of the following domain [26]. Much like the L-type calcium channels, the pore-forming region is the connecting segments of S5 and S6. This region contains four key acidic residues, including glutamate or aspartate [26]. It forms a tetrameric structure composed of the four domains and is lined by the S5–S6 linkers, known as the p-loops [27]. The arrangement of these residues is responsible for the selectivity of T-type calcium channels [26]. More specifically, the selectivity filter is created by two glutamates in domains I and II and two aspartates in domains III and IV [27]. This differs from the selective region of four glutamate residues in L-type calcium channels [21]. Similar to voltage-gated ion channels [6], the S4 segment is composed of a positively charged arginine or lysine residue that serves at the voltage sensor [26].

Although T-type calcium channels are present in embryonic hearts, their role diminishes during the development process [25]. By the time the heart matures, T-type channels are almost undetectable in ventricular myocytes. They are mostly present in the conduction system of the heart [17]. Therefore, they function in the pacemaker role and depolarization of the sinoatrial nodal cells of the adult heart [25]. Abnormal function of these channels can result in Bradycardia as they are the contributors for the pacemaker activity [9]. Of note, it has been shown that the presence of these channels in atrial and ventricular myocytes, where mostly L-type calcium channels are found, results in pathological conditions and dysregulation of the excitation–contraction coupling, leading to abnormal electrical activity in the heart [25]. T-type channels that are erroneously present in adult myocytes have been shown to result in hypertrophied myocytes [28]. This is largely considered to be due to the role of T-type channels in the remodeling of the heart. Furthermore, the inhibition of these channels results in increased fibrosis in the heart due to diastolic dysfunction leading to impaired relaxation [9].

It is to be noted that calcium channels are differentially regulated in the cardiac conduction system and myocardium. For example, *Cacna1c* of Cav1.2, *Cacnb2,* and RyR2 are involved in the plateau phase of the action potential and are highly expressed in cardiomyocytes. In contrast, *Cacnb1* and *Cacnb3* are enriched in the conduction system [29] and T-type channels are enriched in the adult SAN and AV node [30].

## 3. Heart Diseases Associated with Calcium Channels

Calcium homeostasis is crucial to the structural and physiological functions of the heart. More specifically, the calcium influx through the cardiac voltage-gated calcium channels is important for appropriate cardiac function [15]. When there is an upset in this balance, pathological conditions arise, ranging from benign to life-threatening in nature. The function of calcium channels ranges from pacemaker ability to the contractility of the heart. Mutations of calcium channels can cause major dysfunction including spontaneous death without the presence of structural heart disease.

The main reason for disorders that arise from calcium channel dysfunction is the prolongation of cardiac action protentional duration. This dysfunction increases calcium loading in the cells due to increased and prolonged entry of calcium, resulting in reduced diastolic removal of calcium. L-type channels further participate in delayed afterdepolarizations by bringing more calcium in at the same time. Such increased calcium currents contribute to calcium-related arrhythmias [31].

The most prevalent calcium channel present in the adult mammalian cardiovascular system, in particular cardiac myocytes, is Cav1.2 (α1C), a voltage-gated L-type calcium channel [28]. It also has pacemaker activity, specifically in the atrioventricular node. In addition, there are also two voltage-gated T-type calcium channels, Cav3.1 (α1G) and Cav3.2 (α1H), present in conduction and pacemaker cells of cardiac myocytes. They regulate both the sinoatrial and atrioventricular node. There are also other isoforms that are expressed in different cell types of the heart, including the L-type channels Cav1.3 (α1D) and Cav2.3 (α1E) present in cardiac smooth muscle cells [18]. Furthermore, there are calcium store-operated calcium channels present in cardiac fibroblasts [18].

Cav1.2 is present in both the developing and mature hearts and is the primary signal for the contractility of the heart from the excitation-contraction coupling [28]. The inward current driven by this channel is the major contributor to the action potential plateau phase and, therefore, a key mediator of the cardiac action potential [15]. Cav3.1 and Cav3.2 are expressed only in the developing heart given they do not play a role in the myocardial cells briefly after birth. They become localized to the pacemaker in adult hearts [27]. T-type channels have been shown to be expressed in adult hypertrophied cardiomyocytes [9].

Cav1.2 contributes to a major physiological role in the contractile function of the heart. It serves as the primary channel to regulate calcium entry for excitation–contraction coupling [11]. Therefore, knockout models have been shown in the *Cacna1c* (which encodes Cav1.2) gene in the mouse model to lead to major cardiac defects and premature death in embryonic life [9,32]. The channel’s involvement in the cardiac pacemaker activity also results in significant bradycardia and arrhythmias in knockout models [9]. Several channelopathies have been implicated with Cav1.2.

The *Cacna1c* gene differential dysfunction results in varied functional consequences. Given L-type channels are encoded by this gene, it regulates function in the atrioventricular conduction and ventricular myocytes [33]. When it is a gain of abnormal function at the cellular level, calcium currents are increased and it results in QT prolongation (Timothy syndrome). In contrast, when there is a loss of function, short QT intervals result in reduced calcium currents and it can result in Brugada syndrome [34].

## 4. Timothy Syndrome

Timothy syndrome (TS) is a rare childhood disorder that has only been associated with the *Cacna1c* gene [33]. Previously known as ‘heart and hand syndrome’ [35], TS is a rare variant of Long QT syndrome. It is characterized by pronounced invariant long QT interval and webbed toes or fingers (syndactyly). It also has secondary characteristics such as dysmorphic facial features, immune deficiency, cognitive abnormalities, and autism [36]. TS occurs due to a mutation in the L-type calcium channel (Cav1.2) mutation, specifically sporadic missense mutations in G406R, G402,S and GL06R. The mutation implicated is a glycine residue between segment 6 of domain I and the loop between domains I and II, resulting in a gain of function system [33]. Gain of function mutation G406R causes cardiac arrhythmia and long QT, and G402S is specifically associated with decreased channel inactivation, causing a prolonged depolarizing L-type calcium current. The most common cause of death with this syndrome is the onset of cardiac ventricular fibrillation. Most patients do not survive past the first 2–3 years. This syndrome is a multisystem genetic disorder and affects other organs, such as the skeletal system, metabolic system, and the brain along with the ventricular myocytes in the heart, given that Cav1.2 is expressed in other tissues as well besides the heart.

## 5. Brugada Syndrome

Brugada syndrome (BrS) is another channelopathy that can result from a loss of function mutation in the *Cacna1c* gene [33]. It is a rare arrhythmia characterized by a right bundle branch block and ST-segment elevations in the right precordial leads [37]. Patients can either be asymptomatic or develop ventricular fibrillation, being at high risk for sudden cardiac death [37,38,39].

Unlike Timothy Syndrome, Brugada syndrome was diagnosed to have a normal QT interval and, instead, have persistent ST-segment elevation [37,38,39]. Symptomatic BrS patients are more often male adults and have a loss of AP dome and the development of phase 2 re-entry and polymorphic ventricular tachycardia [40,41]. There are three classifications of BrS: BrS3, BrS4 and BrS9 with mutations in *Cacna1c*, *Cacnb2,* and *Cacna2d1*, respectively [37,38,39,42].

On the other hand, the two T-type mammalian channels, Cav3.1 and Cav3.2, carry different functions [28]. Cav3.1 has been shown to affect the heart rate due to its direct role in the acetylcholine-induced relaxation of coronary arteries, whereas Cav3.2 is responsible for pressure overload-induced cardiac hypertrophy [9]. Both channels affect peripheral vascular smooth muscle cell functionality and, therefore, resistance. Cav3.1 channels play a significant role in the generation of pacemaker potentials. The knockout mouse model in the *Cacna1g* gene has a decreased pacemaker activity and atrioventricular conduction [9]. Due to the channel’s role in depolarization in the sinoatrial node and propagation of the impulse, bradycardia also resulted in the knockout model [9]. Cav3.2 channels do not contribute to pacemaker activity as the knockout mouse model in the *Cacna1h* gene resulted in mice with normal heart rates and no arrhythmias [9]. However, Cav3.2 channels have been shown to be involved in the development of cardiac hypertrophy through activation of the calcineurin/NFAT pathway [9]. On the other hand, Cav3.1 channels are suggested to demonstrate a protective role in the development of cardiac hypertrophy through the activation of cGMP production dependent on eNOS activation [9]. Furthermore, Cav3.2 knockout models have shown that those mice develop increased cardiac fibrosis due to impaired relaxation of the coronary vessels [9]. The size of fibrosis, which is typically in the ventricular walls, increases with age and necrosis. The inhibition of these channels plays a protective role in cardiac failure [9].

**Figure 2:**
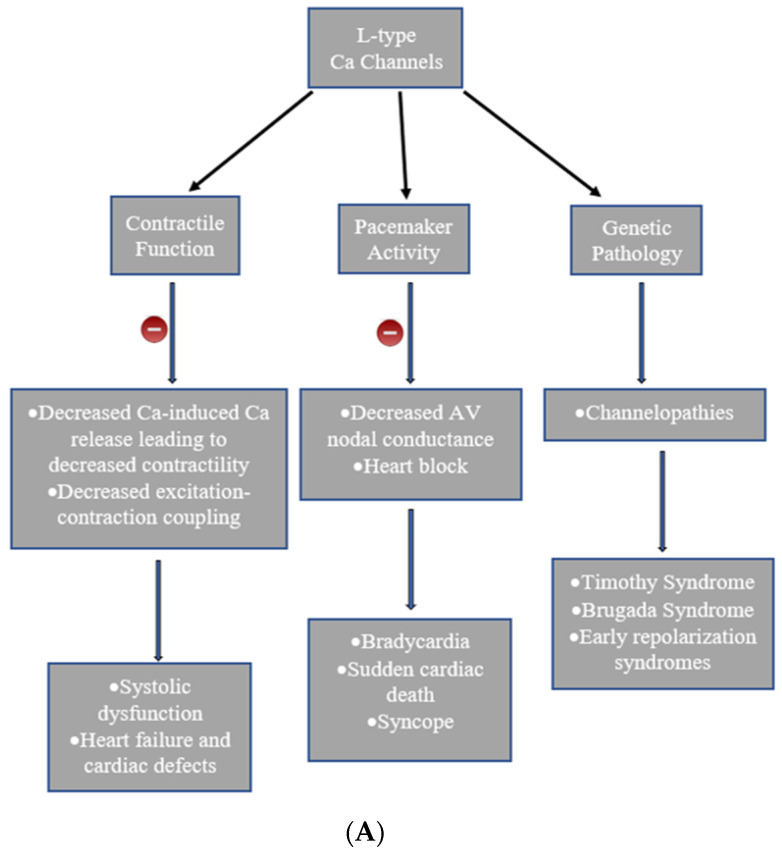

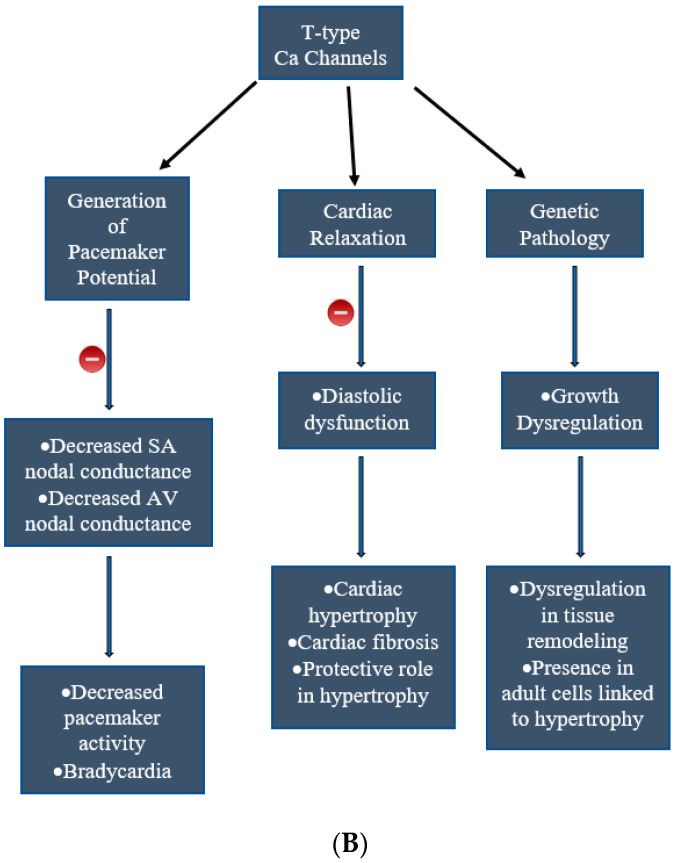
Charts represent the types of calcium channels in the heart, their function and the dysfunctions they are associated with. (**A**). L-Type channels, (**B**). T-type channels.

Currently, 80% of Brugada Syndrome or Short QT Syndrome patients receive a negative result for any of the currently identified genes. Treatment on the other hand is made more complex with the wide variety of nucleotide changes in each disease state. Gene-targeted therapy remains an unlikely path possible in diagnosing and treating these syndromes due to the many mutation manifestations currently known and the many still unknown. An overview of cardiac calcium channels, their functions, and related abnormalities are charted in Figure 2.

Loss of function mutation in the *Cacna1c* gene has also been implicated with early repolarization syndrome, where prominent J waves or J point elevations are seen on an ECG [15]. This can predispose to life-threatening arrhythmias due to accentuation of the action potential [15]. Interestingly, mutations in this channel have also been seen in short QT syndrome. These mutations have been associated with gain-of-function mutations in repolarizing currents [15].

Early repolarization syndrome (ERS) is a type of sudden cardiac death syndrome, inherited and known to be caused by several ion channel dysfunctions. Recent studies have implicated the *Cacna1c*-P817S mutation that causes impaired trafficking of Ca_V_1.2 protein to be leading to ERS phenotypes [42,43].

## 6. Drugs Targeting Calcium Channels

Calcium blocker discoveries date back to the 1960s [44,45], when Godfraind and colleagues discovered the calcium channel antagonistic activities of the compounds lidoflazine, cinnarizine and chlorpromazine. Since then, even though calcium channel blockers (CCBs) have been investigated and used to treat various disorders, there have been questions regarding their safety due to fatalities and arrhythmias associated with some of these drugs [46,47,48]. However, CCBs are still currently used to treat various conditions relating to cardiac function including, but not limited to, atrial arrhythmia, hypertension, tachycardia and angina [49].

CCBs can be divided broadly into two groups: dihydropyridines (DHP) and non-dihydropyridines (non-DHP). These have the same mechanism of action but different effects on cardiac function. Some examples of drugs that are classified as non-DHP mainly including diltiazem and verapamil. Some examples of DHP include: amlodipine, nifedipine, clevidipine, and felodipine. DHPs can cause reflex tachycardia due to their shorter half-life compared to non-DHP, but extended-release formulations have largely alleviated this issue [50]. DHPs are considered to be more selective than non-DHPs, but non-DHPs are more effective at lowering the force and rhythm of cardiac contractions [46]. A list of currently approved CCBs can be found in Table 1. Table 2 lists different types of calcium channels, their location in the heart and the class of drugs they are targeted with [17,28].

Non-DHPs and DHPs prevent calcium ions from entering the cardiac smooth muscle cells when they are depolarizing. This causes arterial vasodilation due to a lower calcium intracellular concentration. Vasodilation leads to lower blood pressure when the blood vessels are wider and allow for more movement of blood, which can help treat angina and hypertension [49]. This has overall negative inotropic and chronotropic effects on the heart, leading to decreased demand for oxygen as well as lower blood pressure. Non-DHPs affect the heart’s conductivity and contractility and affect the sinoatrial node and atrioventricular node [47]. DHPs are mainly used in peripheral vasodilation due to the fact that they have little effect on the myocardium [47]. Below are brief specific descriptions of how these drugs are used in various cardiovascular disorders.

## 7. Angina

Nifedipine is found to have similar properties to the beta-blocker propranolol and relives exertion ischemia by improving exercise wall motion. Beta-blockers can be combined with CCBs, given that beta-blockers have a better effect on heart rate while drugs such as nifedipine preserve cardiac output [48]. Amlodipine also prevents cardiovascular events and has less of an effect on glycemic control than beta-blockers. Hence, CCBs are used for hypertensive patients with stable ischemic heart disease. Verapamil is also useful in reducing ST depression [51]. Diltiazem can be used in stable angina and, similar to the beta-blocker propranolol while also inducing bradycardia, most likely due to blockage of Cav3 channels [20]. CCBs are also used to treat other types of angina such as silent angina, vasospastic angina, and unstable angina along with acute myocardial infarction.

## 8. Arrhythmia

CCBs have antiarrhythmic properties given their capacity to control the firing of the pacemaker of the heart. At the atrioventricular node, they can also reduce conduction velocity, thereby prolonging repolarization. Their action at the AV node interferes with re-entry circuits that cause supraventricular tachycardia, hence making CCBs useful therapeutics to treat such arrhythmias. CCBs fall under class IV arrhythmia drugs. Verapamil and diltiazem given intravenously can reduce ventricular response as per trials [52].

## 9. Hypertension

CCBs do not modify venous tone but can cause arterial dilation and, hence, be used as antihypertensive drugs, especially in combination with other drugs across genders and ages. CCBs reduce resistance at the blood vessels, causing a decrease in blood pressure. Nifedipine reduces blood pressure in hypertensive patients but not in normotensive people. Hence, it is used in combination with other agents such as angiotensin receptor blockers or ACE inhibitors [50].

## 10. Atherosclerosis

CCBs such as amlodipine, lacidipine, and nisoldipine, etc. act as antioxidants as they are lipid-soluble and are used as antiatherosclerosis agents. They reduce the oxidation of low-density lipids and hence prevent their accumulation in the arteries. Long-term clinical trials have suggested that these drugs can help minimize atherosclerosis in hypertensive and coronary heart disease patients [53,54].

Although calcium channel blockers are widely used in treating cardiac disorders, there are several challenges due to limited choices and the drugs not being fully efficient. They are also not specific for specific variants of the calcium channels and the drugs also result in severe adverse effects. Hence, it is of prime importance that future drug research for calcium blockers focuses on targeting specific variants with high efficacy and specificity.

## 11. Future Perspective

With research in animal models, it is now clear that cardiac calcium channels are important in the regulation of heart rate, pacemaking, and cardiac conduction. Several of these channels are well-conserved in humans and it is imperative that human homologs will also have similar properties. However, there need to be further studies to correlate all of the rodent data in humans, which should be carried out in the next couple of decades. While calcium channel blockers are effective drugs against a wide array of cardiac diseases, they have been reported to have various adverse effects such as headaches, lightheadedness, peripheral edema, constipation, and bradycardia, among others. Hence, there is a further need to optimize the pharmacology of these drugs. Moreover, it is unclear what are the long-term effects of these drugs as that is another field to be studied thoroughly in animal models followed by clinical trials. Although we discuss only cardiac channels for the scope of this review, it should be emphasized that they are major players in neuronal conductivity as well as non-cardiac muscle contraction. Given their importance in other fields, and the various disorders and diseases they are involved in, further understanding of calcium channels and calcium channel blocker drug research holds immense potential for the future. Large-scale meta-drug data analysis and ongoing translational research are key to providing further breakthroughs in this field.

## Figures and Tables

**Table 1:** Chart represents classes of calcium channels blockers, their trade names and the disorders they are used for.

Drug	Brand Names	Indications
Non-Dihydropyridine calcium channel blockers		
Diltiazem	Cardizem LA, Dilacor, Tiazac	Hypertension, Angina, Arrythmia
Verapamil	Covera-HS, Verelan PM, Calan	Hypertension, Angina, Arrythmia
Dihydropyridine calcium channel blockers		
Amlodipine	Norvasc	Hypertension, Angina
Felodipine	Plendil	Hypertension
Nifedipine	Adalat, Procardia	Hypertension, Angina
Nicardipine	Cardene	Hypertension, Angina
Nisoldipine	Sular	Hypertension
Isradipine	Dynacirc	Hypertension
Nimdipine	Nimotop	Hypertension

**Table 2:** Chart represents types of calcium channels, their location in the heart and the class of drugs they are targeted with.

Type of Calcium Channel	Location in Myocardial Cell Types	Targeted Drugs
Cav1.2 (L-type)	Ventricular Conduction	DHP
Cav3.1 (T-type)	Pacemaker Conduction	DHP
Cav3.2 (T-type)	Pacemaker Conduction	DHP
Cav2.3 (L-type)	Smooth muscle	DHP and non-DHP
Cav1.3 (L-type)	Smooth muscle	DHP and non-DHP
Calcium store-operated calcium channels	Endothelial cells	None
